# Sirtuin 5‐mediated desuccinylation of PRDX6 inhibits ferroptosis and alleviates sepsis-associated acute kidney injury

**DOI:** 10.1080/13510002.2026.2657075

**Published:** 2026-04-10

**Authors:** Wenlong Lin, Caitao Dong, Qinhong Jiang, Yuanquan Lou, Long Wang, Ziqi He

**Affiliations:** a Department of Anesthesiology, Renmin Hospital of Wuhan University, Wuhan, People's Republic of China; b Department of Urology, Renmin Hospital of Wuhan University, Wuhan, People's Republic of China

**Keywords:** Sepsis-associated acute kidney injury, ferroptosis, SIRT5, PRDX6, Lipid peroxidation, succinylation, desuccinylation, post-translational modification

## Abstract

**Objectives:**

To investigate the role of the SIRT5-PRDX6 axis during the pathogenesis of sepsis-associated acute kidney injury (SA-AKI).

**Methods:**

*In vivo* and *in vitro* sepsis models were established to evaluate oxidative stress and inflammatory responses. High-throughput proteomics analysis identified ferroptosis as a key mechanism underlying SA-AKI. The levels of HMOX1, NQO-1, GPX4, ACSL4, Fe²⁺, IL-1β, TNF-*α*, MDA, and GSH were measured. SIRT5 knockdown/overexpression experiments were performed in HK-2 cells, and SIRT5-deficient mice were used to explore its regulatory role. Co-immunoprecipitation (Co-IP) and site-directed mutagenesis verified SIRT5-PRDX6 interaction and desuccinylation sites.

**Results:**

Ferroptosis was critical in SA-AKI progression. In LPS-induced HK-2 cells, HMOX1, NQO-1, ACSL4, Fe²⁺, IL-1β, TNF-*α*, and MDA were significantly increased, whereas GSH and GPX4 were downregulated. Treatment with ferrostatin-1 (Fer-1) attenuated ferroptosis and oxidative damage. SIRT5 decreased in a time-dependent manner following LPS stimulation. SIRT5 knockdown exacerbated LPS-induced ferroptosis, whereas SIRT5 overexpression suppressed it. SIRT5 activation alleviated AKI in mice, whereas SIRT5 deficiency aggravated it. Mechanistically, SIRT5 desuccinylated PRDX6 at lysine 209, thereby inhibiting inflammatory and oxidative stress responses, attenuating ferroptosis, and ultimately ameliorating renal injury.

**Conclusion:**

The SIRT5-PRDX6 axis regulates SA-AKI pathogenesis by modulating ferroptosis and represents a novel potential therapeutic target.

## Introduction

Sepsis is a life-threatening organ dysfunction caused by a dysregulated host response to infection [1]. A 2020 Global Burden of Disease report published in *The Lancet* estimated approximately 49 million cases of sepsis worldwide in 2017, with a sepsis-related mortality rate of about 22%, accounting for 19% of all global deaths [2]. Sepsis-associated acute kidney injury (SA-AKI) is one of the most prevalent and severe complications of sepsis, associated with poor prognosis and high mortality [3]. A retrospective study involving 146,148 patients in China reported that 47.1% of sepsis patients developed AKI [4]. Therefore, early identification of individuals at risk of SA-AKI and timely initiation of appropriate supportive interventions are critical [5]. Recent studies have utilized biomarkers of renal tubular injury, such as neutrophil gelatinase-associated lipocalin (NGAL), kidney injury molecule-1 (KIM-1), and tissue inhibitor of metalloproteinase-2 (TIMP-2), for early diagnosis of kidney damage [6,7]. However, the pathogenesis of SA-AKI is complex, and further research is needed to fully elucidate its underlying pathophysiological mechanisms [8].

Sirtuins (SIRT1–SIRT7) are an evolutionarily conserved family of NAD⁺-dependent deacetylases, deacylases, and ADP-ribosyltransferases [9]. SIRT5 is predominantly localized in mitochondria and exhibits deacetylase, desuccinylase, deglycosylase, and deglutarylase activities [10]. Among these, its desuccinylase activity significantly influences the structure and function of various proteins and plays a critical role in the development and progression of multiple diseases [11,12]. Succinylation refers to the addition of a succinyl group (─CO─CH₂─CH₂─CO₂H) to a protein lysine residue, neutralizing its positive charge (+1) and conferring a net negative charge (–1) [13]. Moreover, unlike lysine methylation or acetylation, succinylation introduces a relatively large structural moiety (100 Da), and such structural and charge alterations can profoundly affect protein function [14]. Notably, Yu et al. reported that SIRT5 was significantly downregulated in acetaminophen-induced liver injury (AILI), and its depletion exacerbated mitochondrial oxidative stress [15]. Similarly, Li et al. demonstrated that SIRT5 alleviated sepsis-induced acute kidney injury by desuccinylating ATPase inhibitory factor 1, thereby suppressing excessive mitochondrial fission [16].

Ferroptosis is a non-apoptotic form of cell death characterized by iron-dependent accumulation of phospholipid hydroperoxides, ultimately leading to membrane rupture and cell death [17]. Given its involvement in various clinical conditions, including cancer, neurodegeneration, and tissue injury, the mechanisms regulating ferroptosis have garnered increasing attention [18]. Recent studies have highlighted a significant role for ferroptosis in the pathogenesis of several kidney diseases, such as acute kidney injury (AKI), diabetic nephropathy, and renal clear cell carcinoma [19]. Notably, Chen et al. demonstrated that PRDX6 mediates SCLY-independent selenocysteine metabolism to sustain GPX4 expression, thereby suppressing ferroptosis and representing a potential therapeutic target [20].

To date, no study has investigated the role of the SIRT5-PRDX6 axis in the pathogenesis of SA-AKI. In the present study, we found that SIRT5 was significantly downregulated in both *in vivo* and *in vitro* models of SA-AKI. Mechanistically, SIRT5 desuccinylates PRDX6 at lysine 209, counteracting the reduction of GPX4 and thereby attenuating ferroptosis to alleviate kidney injury. Thus, the SIRT5-PRDX6 axis plays a critical role in SA-AKI and may represent a promising avenue for therapeutic intervention.

## Materials and methods

### Animal model

The Laboratory Animal Welfare and Ethics Committee at Renmin Hospital of Wuhan University carefully examined and approved the animal experiment protocols (Issue No. 20250205 A). 6 to 8-week-old male mice were used throughout the study. The Laboratory Animal Center of Renmin Hospital of Wuhan University provided pathogen-free wild-type (WT) C57BL/6J mice. SIRT5^flox/flox^ mice and Cdh16-cre mice were purchased from Saiye Biotechnology Co. (Guangzhou, China). The mouse cadherin 16 (Cdh16) promoter controls the expression of Cre recombinase in Cdh16-cre mice, which is expressed in the renal tubules of adult mice as well as in the epithelial cells of the developing kidney. SIRT5^flox/flox^ mice were crossed with Cdh16-cre mice to produce renal tubular epithelial cell-specific SIRT5-deficient mice. All the mice in this study were humanely euthanized. Briefly, place the mice to be euthanized in a sealed euthanasia device, and release CO_2_to cause the mice to suffocate and die.

Animal experiments were conducted in two parts, and each group in each part comprised five mice (*n* = 5). The experimental groups were structured as follows:

Part I: Sepsis was induced by the cecal ligation and puncture (CLP) procedure to evaluate the impact of RTEC-specific SIRT5 deficiency on renal injury. Laparotomy and intestinal manipulation were performed on animals undergoing sham surgery. Twenty mice were randomly assigned into four groups: (1) WT + Sham group; (2) SIRT5CKO + Sham group; (3) WT + CLP group; (4) SIRT5CKO + CLP group.

Part II: The specific SIRT5 agonist MC3138 (HY-160818, MCE, U.S.A.) was dissolved in DMSO to a concentration of 2 mg/mL and administered intraperitoneally to Pathogen-free wild-type (WT) C57BL/6J mice at a dosage of 20 mg/kg body weight, 30 minutes before the cecal ligation and puncture (CLP) procedure. To assess the therapeutic potential of SIRT5 activation, mice were divided into four groups: (1) Sham group; (2) Sham + MC3138 group; (3) CLP group; (4) CLP + MC3138 group.

### Protein extraction and trypsin digestion

Kidney tissues were harvested from sham-operated (*n* = 5) and CLP (*n* = 5) mice 24 hours post-surgery. The material was put into a 5-mL centrifuge tube after being pulverized into cell powder using liquid nitrogen. A high-intensity ultrasonic processor (Scientz) was then used to sonicate the cell powder for three minutes on ice after adding four volumes of lysis buffer (8 M urea, 1% protease inhibitor cocktail, v/v). The remaining debris was removed by centrifugation at 12,000 g at 4 °C for 10 minutes. Finally, the supernatant was collected, and the protein concentration was determined with the BCA kit according to the manufacturer's instructions. After protein extraction, the sample was slowly added to the final concentration of 20% (w/v) TCA to precipitate protein, then vortexed to mix and incubated for 2 hours at 4 °C. The precipitate was collected by centrifugation at 4500 g for 5 minutes at 4 °C. The precipitated protein was washed with pre-cooled acetone three times and dried for 1 minutes. The protein sample was then redissolved in 200 mM TEAB and ultrasonically dispersed. A 5-mL centrifuge tube was filled with the sample after it had been pulverized into cell powder using liquid nitrogen. The cell powder was then combined with four liters of lysis buffer (8 M urea, 1% protease inhibitor cocktail, v/v) and sonicated for three minutes on ice using a high-intensity ultrasonic processor (Scientz).

### LC-MS/MS analysis

A home-made reversed-phase analytical column (15 cm in length, 100 μm i.d.) was immediately loaded with the dissolved tryptic peptides in solvent A. Solvent A (0.1% formic acid in water, v/v) and solvent B (0.1% formic acid, 80% acetonitrile/in water, v/v) made up the mobile phase. Peptides were separated with the following gradient: 0–14 minutes, 8%–27%B; 14–16 minutes, 27%–36%B; 16–17 minutes, 36%-99%B; 17–20 minutes, 99%B, and all at a constant flow rate of 450 nl/min on a Vanquish Neo UHPLC system (ThermoFisher Scientific). The peptides underwent timsTOF HT mass spectrometry after being exposed to a capillary source. 1.6 kV was the electrospray voltage used. The TOF detector was used to investigate precursors and fragments. Data-independent parallel accumulation serial fragmentation (dia-PASEF) mode was used to run the timsTOF HT. Eight PASEF MS/MS mode MS/MS scans were obtained every cycle, with the complete MS scan set at 100–1700 MS/MS scan range. The MS/MS scan range was set as 425–1025, and the isolation window was set as 25 m/z.

### Databases search and bioinformatic analysis

Spectronaut (v.18) software was used to process the DIA data. Tandem mass spectra were compared to a reverse decoy database concatenated with the Mus_musculus_10090_SP_20231220.fasta (17191 entries). Trypsin/P was identified as a cleavage enzyme that permits up to two missed cleavages. On Cys, carbamidomethyl was designated as a permanent alteration. Variable changes were identified as oxidation on Met and acetylation on the protein *N*-terminal. Protein, peptide, and PSM false discovery rates (FDR) were set at less than 1%.

### Cell culture and intervention

Human proximal tubular epithelial cells (HK-2) were procured from Procell Life Science & Technology Co., Ltd. (Wuhan, China) for these studies. Cells were cultivated in DMEM/F12 medium (Gibco, USA) supplemented with 10% (v/v) fetal bovine serum (FBS, Biological Industries, Israel) and 1% (v/v) penicillin streptomycin (C0222, Beyotime Biotech Inc., China). A humidified incubator at 37 °C with a 5% CO2 atmosphere was used as the culture environment. Culture medium was refreshed daily, and cells were passaged upon reaching 80%–90% confluence. To establish an in vitro model of sepsis-associated kidney injury, HK-2 cells were stimulated with 10 µg/mL lipopolysaccharide (Escherichia coli O111:B4, Cat. #L2630; Sigma, USA).

### Plasmid and cell transfection

Lentiviral (LV) vectors for SIRT5 knockdown (sh-SIRT5) and SIRT5 overexpression (OE-SIRT5), along with the corresponding negative controls, were generated by OBiO Technology Corp., Ltd. (Shanghai, China). Stable transfected cell lines were selected using medium containing 5  μg/mL puromycin (C0222; Beyotime Biotech Inc., China). PRDX6 mutant plasmids were also synthesized by OBiO Technology (Shanghai, China). Cell transfections were performed using Lipofectamine™ 2000 reagent (11668019; Thermo Fisher Scientific Inc., USA) following the manufacturer's protocol.

### Cell viability assay

HK-2 cells were cultured in 96-well plates (100 μL per well) at 37 °C with 5% CO_2_ until the density reached 70%, and Fer-1 (HY-100579, MCE, USA) was treated before harvest. Following a 12-hour incubation period, 100 μl of the CCK8 working solution (BS350B, Biosharp, China) was added to each well, and the mixture was then incubated for two hours at 37 °C. A microplate reader (EnSight, PerkinElmer, USA) was used to measure absorbance at 450 nm.

### ROS measurement

Intracellular reactive oxygen species (ROS) levels were assessed using the fluorescent probe DCFH-DA (C10444, Invitrogen, USA). After washing twice with phosphate-buffered saline (PBS), cells were loaded with 10 μM DCFH-DA and incubated at 37 °C for 20 minutes in the dark. Subsequently, ROS-associated fluorescence was acquired either by flow cytometry (Beckman Coulter, Brea, CA, USA) or by inverted fluorescence microscopy (Olympus IX83, Tokyo, Japan). Relative fluorescence intensity of ROS was quantified with FlowJo v10.6.2 (FlowJo LLC, Ashland, OR, USA) and ImageJ v1.53t (National Institutes of Health, Bethesda, MD, USA), respectively.

### Iron content measurement

For the measurement of Fe^2+^, ferrous iron colorimetric assay kits (E-BC-K772-M) were used (Elabscience, Wuhan, China).

### Mitochondrial membrane potential assay (JC-1)

The JC-1 reagent (E-CK-A301, Elabscience, China) was used to measure mitochondrial membrane potential. Cells were incubated with the staining solution at 37 °C for 20 minutes after the JC-1 assay buffer was diluted 1:10 (v/v) with the JC-1 reagent. The cells were then promptly visualized under the inverted fluorescence microscopy (Olympus IX83, Tokyo, Japan).

### Western blotting

RIPA lysis buffer was used to extract protein from the kidney and cells (Solarbio, Beijing, China). The proteins were separated by SDS-PAGE, then moved to PVDF membranes (1620177, BIO-RAD, USA), blocked for 15 minutes with rapid blocking buffer (PS108P, Epizyme, China), washed four times with TBST, and incubated with primary antibodies for an entire night at 4 °C. Antibodies used included anti-HMOX1 (1:5000, 10701-1-AP, Proteintech), anti-NQO-1 (1:5000, 67240-1-lg, Proteintech), anti-SIRT5 (1:1000, 8782, Cell Signaling Technology), anti-pan-succinyl-lysine (1:1000, PTM-419, PTM Biolabs), anti-GPX4 (1:1000, A21440, Abclonal), anti-ACSL4 (1:1000, A20414, Abclonal), anti-IL-1β (1:5000, 26048-1-AP, Proteintech), anti-TNF-*α* (1:2000, 17590-1-AP, Proteintech), anti-PRDX6 (1:5000, 13585-1-AP, 1:5000, 67499-1-Ig, Proteintech), anti-Cleaved Caspase-3 (1:2000, 87055-4-RR, Proteintech), anti-GSDMD (1:1000, 20770-1-AP, Proteintech), anti-*p*-RIPK3 (1:1000, 87148-1-RR, Proteintech), anti-*p*-RIPK1 (1:1000, 66854-1-Ig, Proteintech), anti-Cleaved PARP-1 (1:1000, 60555-Ig, Proteintech), and anti-*β*-tubulin (1:1000, GB11017-100, Servicebio). The secondary antibodies were then incubated for 1 hour at room temperature with goat anti-rabbit or goat anti-mouse antibodies (1:10000, GB23301, 1:10000, GB23303, Servicebio). An Odyssey dual-color infrared laser imager (LI-COR, USA) and a ChemiDoc XRS system (BIO-RAD, USA) were used to examine the relative expression level of each protein. ImageJ software (ImageJ 1.51j8 version, USA) was used to analyze the gray value.

### Quantitative real-time reverse transcription PCR (qRT-PCR)

As directed by the manufacturer, total RNA was extracted from the treated cells after cell intervention using the TRIzol reagent (15596026, ThermoFisher, USA). The Hifair III 1 st Strand cDNA Synthesis Kit (gDNA digester plus) (11139ES60, Yeasen, China) was then used to synthesize cDNA from 2 μl of RNA. qRT-PCR was configured using the Hieff UNICON Universal Blue qPCR SYBR Green Master Mix (11184ES08, Yeasen, China) on a LightCycler480 (Roche Diagnostics, USA). The normalization reference was GAPDH, and the 2^−∆∆Ct^ technique was used to calculate relative expression changes. The primers used are shown in Table 1.

**Table 1.** Sequences of the relative primers for RT–qPCR.

Gene name	Accession code	Forward primer	Reverse primer
GAPDH	NM_002046.7	GTCTCCTCTGACTTCAACAGCG	ACCACCCTGTTGCTGTAGCCAA
GPX4	NM_002085.5	ACAAGAACGGCTGCGTGGTGAA	GCCACACACTTGTGGAGCTAGA
ACSL4	NM_004458.3	GCTATCTCCTCAGACACACCGA	AGGTGCTCCAACTCTGCCAGTA

### Detection of LDH, T-AOC, SOD, MDA, and GSH

The levels of LDH, T-AOC, and SOD were measured using the LDH assay kit (A020-1-2), T-AOC assay kit (A015-2-1), and SOD assay kit (A001-3-2) according to the manufacturer's instructions (Jiancheng, Nanjing, China). A microplate reader was used to measure the absorbance at 450 nm (LDH), 405 nm (T-AOC), and 450 nm (SOD). The levels of MDA and GSH were measured using the MDA assay kit (JL-T0761) and the GSH assay kit (JL-T0906) strictly following the manufacturer's instructions (Jonlnbio, Shanghai, China). The wavelength for measuring GSH is 412 nm. As for MDA, the content is calculated by simultaneously measuring the absorbance at 600 nm and using the difference between the absorbance at 532 and 600 nm to determine the content of MDA.

### Enzyme-linked immunosorbent assay (ELISA)

Concentrations of IL-1β (JL13662) and TNF-*α* (JL10208) were detected by ELISA kits according to the manufacturer's protocol (Jonlnbio, Shanghai, China). Iron concentration (A039-2-1) was detected by ELISA kits according to the manufacturer's protocol (Jiancheng, Nanjing, China). The measurement of Cleaved caspase-3 (Ab220655) and Cleaved PARP-1 (Ab317545) was detected by ELISA kits according to the manufacturer's protocol (Abcam, Paris, France).

### Blood urea nitrogen (BUN) and serum creatinine (Scr)

BUN and Scr levels were quantitatively examined 24 hours after CLP using an automatic biochemical analysis instrument (Chemray 240, Rayto, Shenzhen, China) according to the manufacturer's instructions.

### Histology and immunohistofluorescence(IHF)

Periodic acid-Schiff staining (PAS) and hematoxylin and eosin staining (HE) were carried out after fresh mouse kidney cortex tissue had been fixed with 4% (v/v) paraformaldehyde for around 24 hours. After that, observation was conducted using an optical microscope (Zeiss, LSM780, Tulingen, Germany). Five random fields of vision were chosen from each of the five mouse parts that were used in each group. The histological assessment of renal damage includes tubular casts, renal tubule hemorrhage, loss of the tubule brush border, inflammatory infiltration, and obvious necrosis in the cortex. For renal tissue immunofluorescence staining, kidney tissues were collected, fixed, and embedded in paraffin. After sectioning the paraffin-embedded tissue block and soaking it in xylene for half an hour, an ethanol gradient solution was used to dewax it. The slices were treated for 12 hours at 4 °C with antibodies against KIM-1 and NGAL after antigen restoration. Secondary antibodies were added to the sections and incubated at room temperature for an hour. The tissue slices were viewed under a positive fluorescence microscope after being washed, stained, counterstained, dehydrated, and sealed.

### Immunofluorescence staining

HK-2 cells were fixed in 4% (v/v) paraformaldehyde (Beyotime, Shanghai, China) for 15 min, 0.1% (v/v) Triton X-100 (T8200, Solarbio, China) for 10 min, and 1% (v/v) BSA (ST2249, Beyotime, China) at room temperature for 1 hour. Primary antibodies against SIRT5(1:1000, 67257-1-Ig, Proteintech) and PRDX6(1:2000, 13585-1-AP, Proteintech) were applied overnight at 4 °C. The cells were rinsed three times with PBS before being treated with goat anti-rabbit (1:10000, GB23301, Servicebio) and goat anti-mouse secondary antibodies (1:10000, GB23303, Servicebio) for one hour at 37 °C. The nuclei were then stained with DAPI (Beyotime, Shanghai, China) at room temperature for 10 minutes. The images were captured using a fluorescence microscope (Olympus IX83, Tokyo, Japan).

### Immunohistofluorescence staining

Kidney tissues were gathered, fixed in 4% (v/v) paraformaldehyde (Beyotime, Shanghai, China), and embedded in paraffin. The paraffin-embedded tissue block was sectioned, treated with xylene for 30 minutes, and then dewaxed using an ethanol gradient solution. Following antigen repair, the sections were treated with antibodies to 4-HNE (Ab46545, 1:100, Abcam). Secondary antibodies were added to the sections and incubated at room temperature for an hour. The tissue sections were viewed under a positive fluorescence microscope.

### Immunoprecipitation and coimmunoprecipitation (Co-IP)

To generate protein lysates, IP lysis buffer (Beyotime, Shanghai, China) was used to lyse cells and kidney tissues, and they were centrifuged at 12,000 × g for 10 minutes at 4 °C. The lysates were treated with protein A + G agarose beads (Beyotime, Shanghai, China) and the relevant primary antibody for a whole night at 4 °C. To extract the proteins, the beads were rinsed three times with IP lysis solution and then boiled in 2 ×  loading buffer at 95 °C for 5 minutes. Finally, the proteins underwent immunoblot analysis.

### Lipid ROS assay

After 12 hours of incubation, cells were cultured in medium containing 5 µM C11 BODIPY 581/591 (Cat. No. D3861; Invitrogen, USA) for 30 minutes. Cells were washed three times with PBS, maintained in 2 mL fresh culture medium, and then promptly visualized under the inverted fluorescence microscopy (Olympus IX83, Tokyo, Japan).

### Measurement of the activity of peroxidase and iPLA2

The activity of peroxidase was measured using the commercial assay kit (BC0095) according to the manufacturer's instructions (Solarbio, Beijing, China). Record the absorbance values A1 at 30 seconds under 470 nm and A2 after 1 minute and 30 seconds. Calculate the difference between A1 and A2 to determine the activity of peroxidase. At the same time, the activity of iPLA2 was measured using the commercial assay kit (MM-63237H2) according to the manufacturer's instructions (Meimian, Jiangsu, China). The absorbance (OD value) was measured at a wavelength of 450 nm using an enzyme detector, and the sample activity was calculated.

### Statistical analysis

GraphPad Prism 10.3.1 (GraphPad Software, USA) was used for all statistical analyzes. Data are expressed as mean  ±  standard deviation (SD). Normality of all the data sets was first assessed using the Shapiro‒Wilk test. Student’s t-test was applied for comparisons between two groups, and one-way analysis of variance (ANOVA) followed by Tukey's multiple-comparison test was used to assess differences among multiple groups. A *P* value < 0.05 was considered statistically significant.

## Results

### Sepsis triggered acute damage to the kidneys

**Figure 1.**
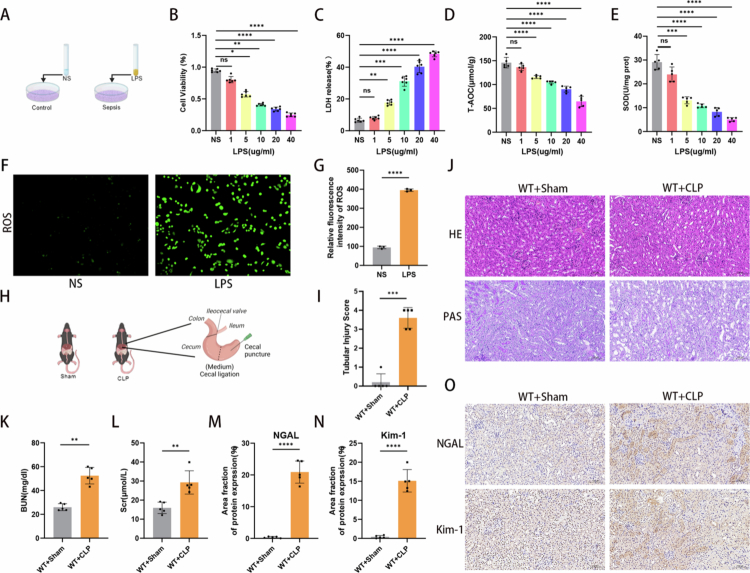
Sepsis-triggered acute damage to the kidneys. (A) Schematic diagram of *in vivo* models of SA-AKI. HK-2 cells were treated with varying concentrations of LPS (0, 1, 5, 10, 20, and 40 μg/ml) and incubated for 12 hours. (B) Cell viability was detected using the Cell Counting Kit-8 (CCK-8) assay (*n* = 6). (C–E) Levels of LDH release (*n* = 6), T-AOC, and SOD under varying concentrations of LPS (*n* = 5). (F) Intracellular reactive oxygen species (ROS) production was evaluated using DCFH-DA fluorescence staining. (G) Relative fluorescence intensity of ROS in the LPS group compared with the NC group (*n* = 3). (H) Schematic diagram of the *in vivo* SA-AKI model established by cecal ligation and puncture (CLP) surgery in wild-type mice. (I) Tubular injury scores based on H&E and PAS staining (*n* = 5). (J) Representative images of H&E and PAS staining of the kidney cortex in the WT + CLP and WT + sham groups. (K) Blood urea nitrogen (BUN) levels at 24 hours after CLP surgery (*n* = 5 mice per group). (L) Serum creatinine (Scr) levels at 24 hours after CLP surgery (*n* = 5 mice per group). (M–N) Bar graphs showing the area fraction of NGAL and KIM-1 expression (*n* = 5). (O) Immunohistochemical staining of NGAL and KIM-1 protein expression in the WT + CLP and WT + sham groups. Representative images are shown. Data are presented as mean ± SD. Statistical significance is indicated as *P < 0.05, **P < 0.01, **P < 0.001, ****P < 0.0001; ns, not significant. One-way ANOVA with Tukey test analysis and two-tailed Studen's *t* test were used for statistical analysis.

To assess the effects of LPS on HK-2 cells and establish an *in vitro* model of renal tubular epithelial cell (RTEC) injury, cells were treated with increasing concentrations of LPS (0, 1, 5, 10, 20, and 40 μg/ml) for 12 hours (Figure 1A). LPS exerted a dose-dependent cytotoxic effect on HK-2 cells, with cell viability significantly reduced following LPS treatment (Figure 1B). Markers of oxidative and antioxidative status, including LDH, T-AOC, and SOD, were then assessed. Compared with the control group, the LPS group showed markedly elevated LDH release (Figure 1C), whereas T-AOC and SOD levels were significantly decreased (Figure 1D and E). Based on the dose-response curves, 10 μg/ml was selected for subsequent experiments as it induced substantial injury. Intracellular reactive oxygen species (ROS) production was evaluated using DCFH-DA fluorescence staining, which revealed that LPS dramatically increased ROS levels compared with the control group (Figure 1F and G). For *in vivo* studies, the SA-AKI model was established by cecal ligation and puncture (CLP), as illustrated in Figure 1H. Serum levels of blood urea nitrogen (BUN) and creatinine (Scr) in CLP-treated mice at 24 hours post-surgery were significantly higher than those in the sham group (Figure 1K and L). Histopathological analysis revealed severe renal injury in CLP mice. Periodic acid–Schiff (PAS) staining showed marked disruption of the proximal tubular brush border in the WT + CLP group, with substantial reduction in apical membrane staining intensity (Figure 1I and J). Furthermore, protein expression levels of KIM-1 and NGAL were dramatically elevated in the WT + CLP group compared with the WT + Sham group (Figure 1M–O). Collectively, these results suggest that sepsis may induce acute kidney injury.

### Quantitative proteomic analysis identified ferroptosis-related pathways in SA-AKI

**Figure 2.**
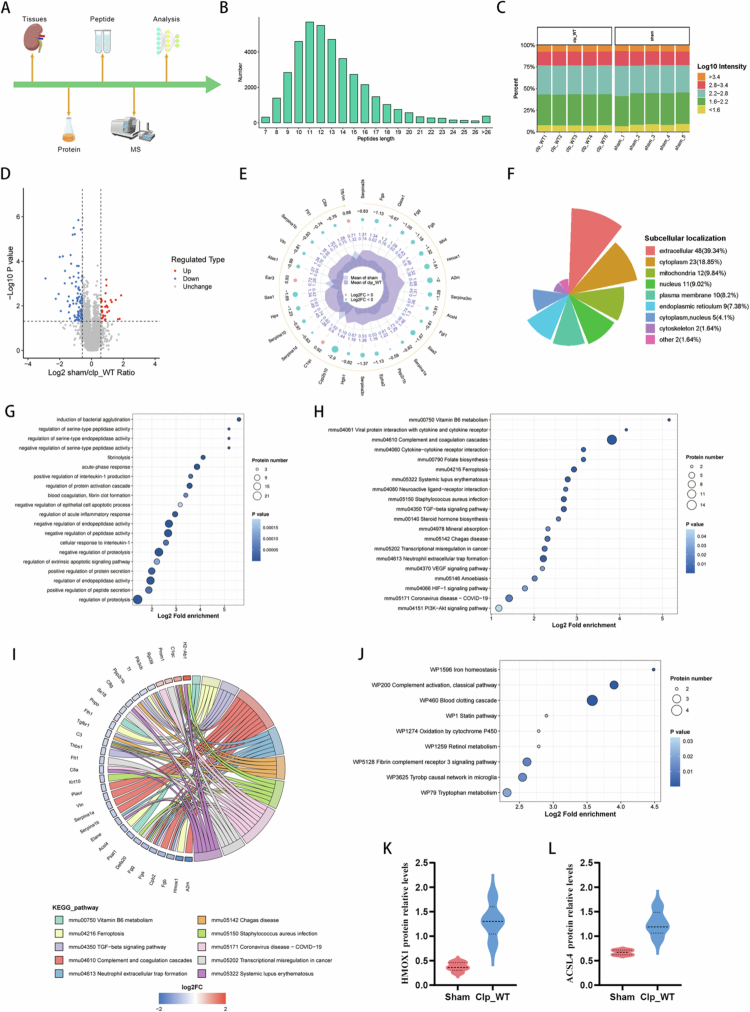
Quantitative proteomic analysis identified mechanisms of sepsis-associated acute kidney injury. (A) Experimental workflow for quantitative proteomic analysis. (B) Distribution of tryptic peptide lengths. (C) Distribution bar chart of the WT + CLP and WT + sham groups. (D) Volcano plot of differentially expressed proteins (DEPs). Proteins with *P* < 0.05 and fold-change ≥ 1.50 are marked in red; proteins with *P* < 0.05 and fold-change ≤ 0.67 are marked in blue. (E) Differential protein radar chart of the WT + CLP and WT + sham groups. (F) Subcellular localization of DEPs. (G) GO-based enrichment analysis of DEPs. (H) Bubble chart of KEGG pathway analysis of DEPs. (I) Chord diagram of KEGG pathway analysis of DEPs. (J) Bubble chart of WikiPathways analysis of DEPs. (K–L) Relative protein levels of HMOX1 and ACSL4 in the WT + CLP and WT + sham groups from quantitative proteomic analysis.

To systematically characterize the renal protein landscape following sepsis, quantitative proteomics analysis was performed (Figure 2A). The majority of identified peptide fragments ranged from 7 to 20 amino acids in length (Figure 2B), and the distribution and variation in protein intensity values between samples were minimal (Figure 2C), indicating that the proteomic data were reliable. Significantly differentially expressed proteins (DEPs) were defined using screening criteria of *P* < 0.05 and a fold-change ≥1.50 or ≤0.67 for subsequent bioinformatic analysis (Figure 2D). Subcellular localization analysis revealed that the majority of DEPs originated from the extracellular region (*n* = 48, 39.34%), cytoplasm (*n* = 23, 18.85%), and mitochondria (*n* = 12, 9.84%) (Figure 2F). To elucidate key pathways involved in SA-AKI, enrichment analysis was performed using Gene Ontology (GO) classification, Kyoto Encyclopedia of Genes and Genomes (KEGG) pathway, and WikiPathways. GO annotation analysis showed that DEPs were predominantly enriched in acute inflammatory response and cellular response to interleukin-1 (Figure 2G). KEGG enrichment analysis indicated that DEPs tended to be enriched in the ferroptosis pathway (Figure 2H and I). Notably, WikiPathways enrichment revealed marked enrichment of iron homeostasis (Figure 2J), directly implicating iron imbalance as a key pathological process in sepsis-induced kidney injury. Furthermore, the differential protein radar chart showed that expression levels of HMOX1 and ACSL4 were significantly increased in the WT + CLP group (Figure 2E, K, and L), suggesting that SA-AKI may be associated with oxidative stress and ferroptosis.

### Fer-1 attenuated LPS-induced ferroptotic injury and inflammatory response in HK-2 cells

**Figure 3.**
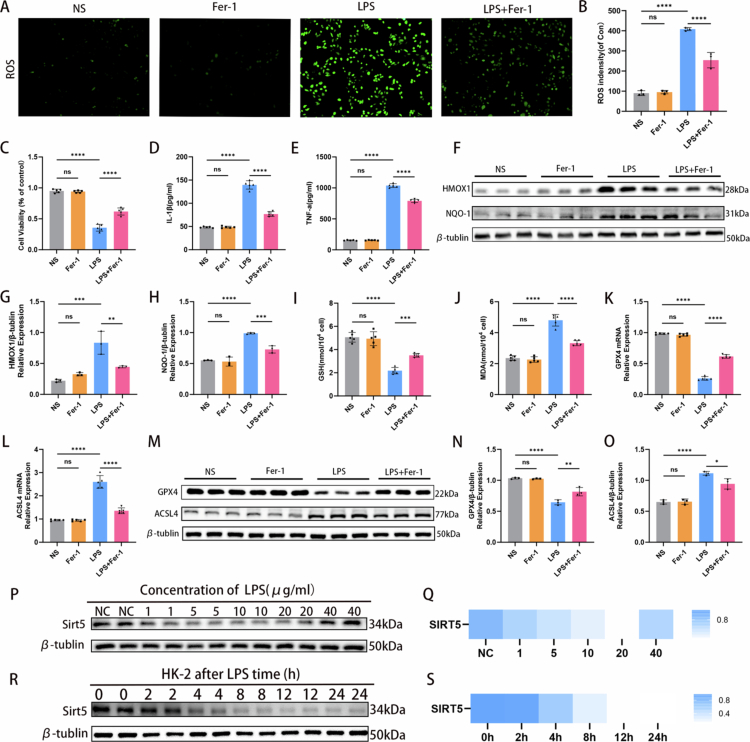
Fer-1 attenuated LPS-induced ferroptotic injury and inflammatory response in HK-2 cells. (A) ROS production was evaluated using DCFH-DA fluorescence staining; brighter green indicates higher cellular ROS levels. (B) Relative fluorescence intensity of ROS in four groups (*n* = 3). (C) Cell viability was detected using the CCK-8 assay (*n* = 5). (D–E) Bar graphs showing levels of the inflammatory cytokines interleukin-1β (IL-1β) and tumor necrosis factor-*α* (TNF-*α*) in four groups (*n* = 5). (F–H) Western blotting results showing protein expression levels of HMOX1 and NQO-1 in four groups. Bar graphs show relative protein expression (*n* = 3). (I–J) Bar graphs showing GSH and MDA levels in four groups (*n* = 5). (K–L) mRNA expression of GPX4 and ACSL4 in HK-2 cells determined by qRT‒PCR (*n* = 5). (M–O) Western blotting results showing protein expression levels of GPX4 and ACSL4 in four groups. Bar graphs show relative protein expression (*n* = 3). (P–Q) Protein expression levels of SIRT5 in HK-2 cells treated with varying concentrations of LPS (0, 1, 5, 10, 20, and 40 μg/ml) (*n* = 3). (R–S) Protein expression levels of SIRT5 in HK-2 cells over time after sustained treatment with 10 μg/ml LPS (*n* = 3). Data are presented as mean ± SD. Statistical significance is indicated as *P < 0.05, **P < 0.01, ***P < 0.001, ****P < 0.0001; ns, not significant. One-way ANOVA with Tukey's test analysis and two-tailed Student's *t* test were used for statistical analysis.

Our proteomic analysis identified ferroptosis as a key pathway in the pathogenesis of SA-AKI. To further explore the role of ferroptotic injury, we investigated the effect of ferrostatin-1 (Fer-1), a specific ferroptosis inhibitor, on LPS-induced HK-2 cell damage. Reactive oxygen species (ROS) production induced by LPS was significantly reduced following Fer-1 treatment (Figure 3A and B). Compared with the LPS group, the LPS + Fer-1 group exhibited increased HK-2 cell viability (Figure 3C) and decreased levels of the inflammatory cytokines IL-1β and TNF-*α* (Figure 3D and E). Additionally, expression levels of the anti-oxidative response proteins HMOX1 and NQO-1 were downregulated upon ferroptosis inhibition (Figure 3F–H). Ferroptosis-associated biomarkers were also assessed. LPS-induced elevation of MDA and reduction of GSH were both attenuated by Fer-1 treatment (Figure 3I and J). Furthermore, Fer-1 administration resulted in upregulated protein and transcript levels of GPX4, and downregulated protein and transcript levels of ACSL4 (Figure 3K–O). Collectively, these results demonstrate that ferroptosis plays a crucial role in the pathogenesis of SA-AKI.

Previous studies have shown that SIRT5 inhibits hepatocellular carcinoma development by suppressing peroxisomal oxidative stress via ACOX1 desuccinylation [21], and that SIRT5 overexpression attenuates cisplatin-induced apoptosis and mitochondrial injury in human renal tubular epithelial cells [22]. However, the dynamic alterations and mechanistic basis of SIRT5 in SA-AKI remained largely unknown. Therefore, we investigated whether SIRT5 exerts a protective role in SA-AKI by desuccinylating and regulating ferroptosis-related proteins. We first examined SIRT5 protein expression in HK-2 cells treated with increasing concentrations of LPS (0, 1, 5, 10, 20, and 40 μg/ml), and observed a dose-dependent decrease (Figure 3P and Q). Similarly, SIRT5 protein expression gradually decreased over time following sustained LPS treatment (Figure 3R and S).

### SIRT5 mediated LPS-induced ferroptosis*in vitro*model of SA-AKI

**Figure 4.**
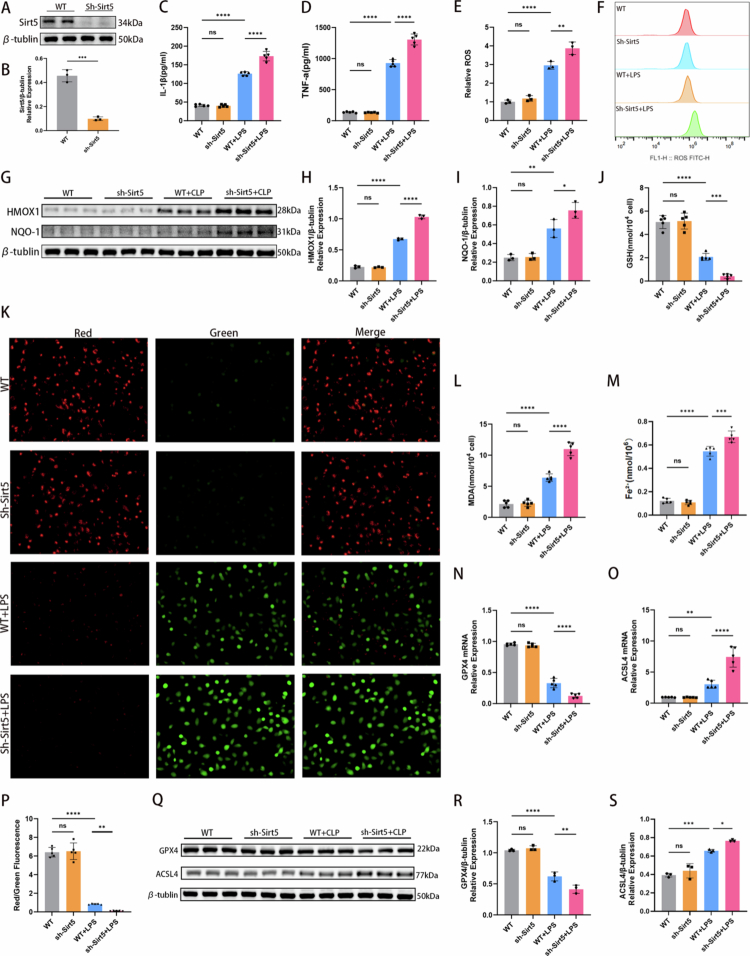
SIRT5 mediated LPS-induced ferroptosis *in vitro* model of SA-AKI. (A–B) SIRT5 knockdown efficiency was determined by western blotting in HK-2 cells. Representative images from three independent experiments are shown (*n* = 3). SIRT5 levels were normalized to *β*-tubulin. (C–D) Bar graphs showing IL-1β and TNF-*α* levels in four groups (*n* = 5). (E) Relative fluorescence intensity of ROS in four groups (*n* = 3). (F) Flow cytometric analysis showing cellular ROS levels in four groups. (G–I) Western blotting results showing protein expression levels of HMOX1 and NQO-1. Bar graphs show relative protein expression (*n* = 3). (J, L) Bar graphs showing GSH and MDA levels in four groups (*n* = 5). (K) Mitochondrial membrane potential of HK-2 cells was assessed using the JC-1 assay. (M) Cellular Fe²⁺ content in four groups was detected using an iron assay kit (*n* = 5). (N–O) mRNA expression of GPX4 and ACSL4 in HK-2 cells in four groups determined by qRT‒PCR (*n* = 5). (P) Quantitative analysis of mitochondrial membrane potential (*n* = 5). A lower red/green fluorescence ratio indicates worse mitochondrial membrane potential. (Q–S) Western blotting results showing protein expression levels of GPX4 and ACSL4 in four groups. Bar graphs show relative protein expression (*n* = 3). Data are presented as mean ± SD. Statistical significance is indicated as *P < 0.05, **P < 0.01, ***P < 0.001, ****P < 0.0001; ns, not significant. One-way ANOVA with Tukey's test analysis and two-tailed Student's *t* test were used for statistical analysis.

To further investigate the role of SIRT5 in SA-AKI, lentiviral (LV) vectors containing small interfering RNA (siRNA) targeting SIRT5 (sh-Sirt5) or a negative control (NC) were used to knock down SIRT5 expression in HK-2 cells (Figure 4A and B). Depletion of SIRT5 significantly exacerbated the inflammatory response induced by LPS. Although LPS stimulation induced substantial secretion of IL-1β and TNF-*α* in the NC + LPS group, this effect was markedly potentiated in the sh-Sirt5 + LPS group (Figure 4C and D). Similarly, LPS treatment robustly increased ROS generation, and ROS levels were further elevated upon SIRT5 knockdown (Figure 4E and F). Protein expression levels of the antioxidant factors HMOX1 and NQO-1 were also significantly increased in the sh-Sirt5 + LPS group compared with the NC + LPS group (Figure 4G–I). Mitochondria are key organelles for ROS production and ferroptosis initiation. The sh-Sirt5 + LPS group exhibited a more severe reduction in mitochondrial membrane potential compared with the NC + LPS group (Figure 4K and P), indicating that SIRT5 loss impaired the cellular capacity to counteract oxidative stress. In addition, MDA content was significantly higher, while GSH levels were more markedly depleted in the sh-Sirt5 + LPS group than in the NC + LPS group (Figure 4J and L). Given that iron metabolism imbalance is a hallmark of ferroptosis, we also measured Fe²⁺ content, which was elevated in the sh-Sirt5 + LPS group compared with the NC + LPS group (Figure 4M). Furthermore, LPS stimulation significantly upregulated ACSL4 expression and downregulated GPX4 expression. In the sh-Sirt5 + LPS group, the suppression of GPX4 was more pronounced, and the upregulation of ACSL4 was further enhanced, at both protein and mRNA levels, compared with the NC + LPS group (Figure 4N, O, Q–S). Collectively, these results indicate that SIRT5 deficiency accelerates and exacerbates LPS-induced ferroptosis.

### SIRT5 overexpression inhibited LPS-induced ferroptosis *in vitro* model of SA-AKI

**Figure 5.**
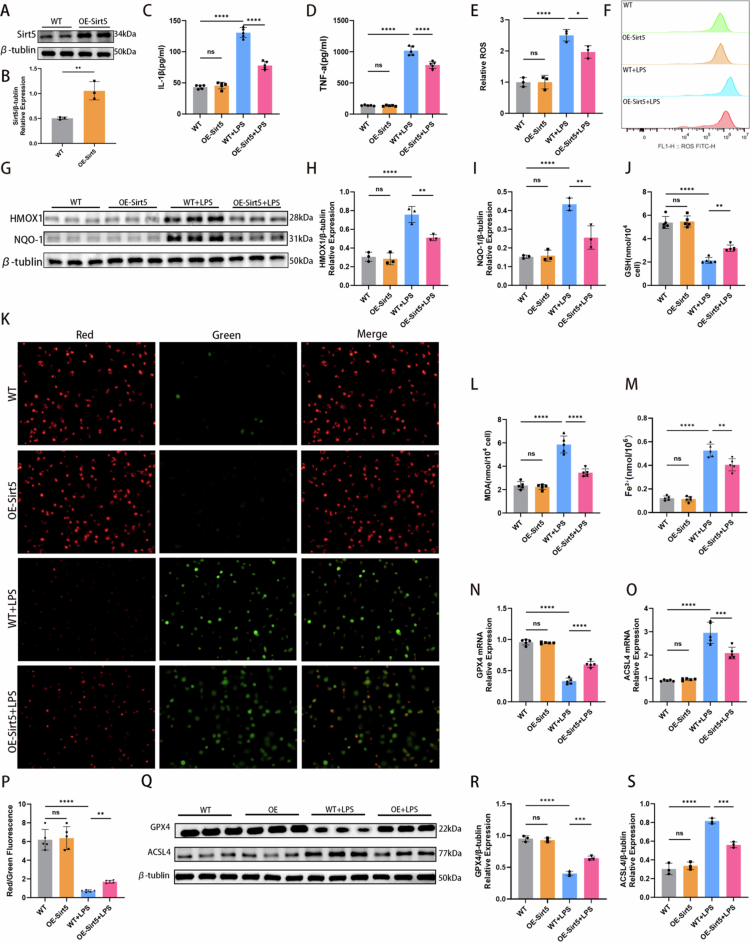
SIRT5 overexpression inhibited LPS-induced ferroptosis *in vitro* model of SA-AKI. (A–B) SIRT5 overexpression efficiency was determined by western blotting in HK-2 cells. Representative images from three independent experiments are shown (*n* = 3). SIRT5 levels were normalized to *β*-tubulin. (C–D) Bar graphs showing IL-1β and TNF-*α* levels in four groups (*n* = 5). (E) Relative fluorescence intensity of ROS in four groups (*n* = 3). (F) Flow cytometric analysis showing cellular ROS levels in four groups. (G–I) Western blotting results showing protein expression levels of HMOX1 and NQO-1. Bar graphs show relative protein expression (*n* = 3). (J, L) Bar graphs showing GSH and MDA levels in four groups (*n* = 5). (K) Mitochondrial membrane potential of HK-2 cells was assessed using the JC-1 assay. (M) Cellular Fe²⁺ content in four groups was detected using an iron assay kit (*n* = 5). (N–O) mRNA expression of GPX4 and ACSL4 in HK-2 cells in four groups determined by qRT‒PCR (*n* = 5). (P) Quantitative analysis of mitochondrial membrane potential (*n* = 5). (Q–S) Western blotting results showing protein expression levels of GPX4 and ACSL4 in four groups. Bar graphs show relative protein expression (*n* = 3). Data are presented as mean ± SD. Statistical significance is indicated as *P < 0.05, **P < 0.01, ***P < 0.001, ****P < 0.0001; ns, not significant. One-way ANOVA with Tukey's test analysis and two-tailed Student's *t* test were used for statistical analysis.

To investigate the effect of SIRT5 overexpression, lentiviral (LV) vectors containing SIRT5 overexpression plasmids were used in HK-2 cells (Figure 5A and B). In the OE-Sirt5 + LPS group, LPS-induced secretion of IL-1β and TNF-*α* was significantly suppressed compared with the NC + LPS group (Figure 5C and D), indicating that SIRT5 overexpression mitigated the inflammatory response in SA-AKI. Additionally, ROS levels were reduced (Figure 5E and F), and protein expression levels of HMOX1 and NQO-1 were significantly lower in the OE-Sirt5 + LPS group than in the NC + LPS group (Figure 5G–I). SIRT5 overexpression also contributed to restoration of mitochondrial membrane potential compared with the NC+LPS group (Figure 5K and P). Notably, MDA and Fe²⁺ contents were significantly lower in the OE-Sirt5 + LPS group than in the NC + LPS group (Figure 5L and M), whereas GSH levels were better maintained (Figure 5J). Furthermore, SIRT5 overexpression effectively restored GPX4 expression at both mRNA and protein levels and reduced ACSL4 expression (Figure 5N, O, Q–S), suggesting that SIRT5 overexpression inhibited LPS-induced ferroptosis.

To further investigate whether the protective effect of SIRT5 on renal tubular epithelial cells (RTECs) involves other forms of programmed cell death, key molecular markers of apoptosis, pyroptosis, and necroptosis were examined. Western blot analysis showed that LPS stimulation significantly upregulated cleaved caspase-3, cleaved PARP-1, GSDMD, and phosphorylated RIPK1/RIPK3 in HK-2 cells. However, SIRT5 overexpression (OE-Sirt5) did not significantly alter the expression of these markers. No significant differences in cleaved caspase-3 or cleaved PARP-1 levels were observed between the OE-Sirt5 + LPS and NC + LPS groups (Fig. S1, Supporting Information). These results indicate that the protective effects of SIRT5 activation are primarily mediated through suppression of ferroptosis.

### SIRT5 deficiency aggravated acute kidney injury in an *in vivo* model of SA-AKI

**Figure 6.**
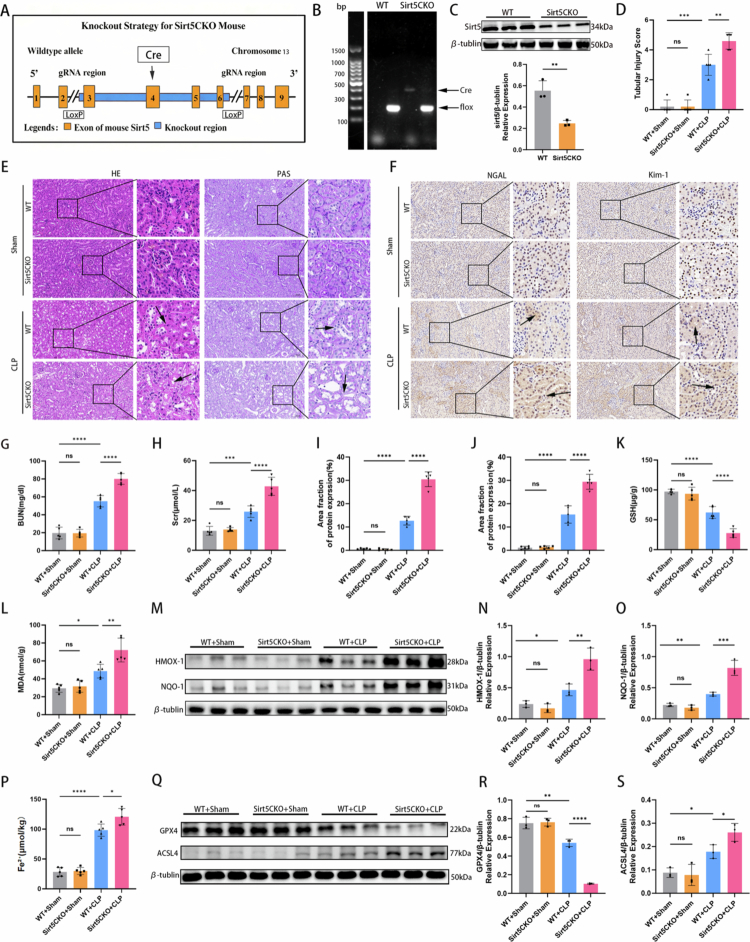
SIRT5 deficiency aggravated acute kidney injury in an *in vivo* model of SA-AKI. (A) Schematic diagram of SIRT5 conditional knockout (Sirt5CKO) mice. (B–C) Absence of SIRT5 in the kidneys of Sirt5CKO mice was confirmed by DNA genotyping and western blot analysis (*n* = 3). (D) Tubular injury scores based on H&E and PAS staining in four groups (*n* = 5). (E) Pathological observation of kidney tissue in four groups. H&E and PAS staining of the kidney cortex. Black arrows indicate necrotic tubular epithelial cells and brush border attenuation. Representative images are shown. (F) Immunohistochemical (IHC) staining of NGAL and KIM-1 in kidney tissues of the four groups. Black arrows highlight positive expression. Representative images are shown. (G–H) BUN and Scr levels at 24 hours after CLP surgery (*n* = 5 mice per group). (I–J) Bar graphs showing the area fraction of NGAL and KIM-1 expression (*n* = 5). (K–L) Bar graphs showing GSH and MDA levels in four groups (*n* = 5). (M–O) Western blotting results showing protein expression levels of HMOX1 and NQO-1 in kidney homogenates. Bar graphs show relative protein expression (*n* = 3). (P) Bar graph showing Fe²⁺ content in kidney homogenates of the four groups (*n* = 5). (Q–S) Western blotting results showing protein expression levels of GPX4 and ACSL4 in kidney homogenates. Bar graphs show relative protein expression (*n* = 3). Data are presented as mean ± SD. Statistical significance is indicated as *P < 0.05, **P < 0.01, ***P < 0.001, ****P < 0.0001; ns, not significant. One-way ANOVA with Tukey's test analysis and two-tailed Student's *t* test were used for statistical analysis.

To further investigate the role of SIRT5 in SA-AKI pathogenesis, SIRT5 conditional knockout (Sirt5CKO) mice were generated (Figure 6A). Absence of SIRT5 in the kidneys of Sirt5CKO mice was confirmed by DNA genotyping and western blot analysis (Figure 6B–C). Hematoxylin and eosin (H&E) and PAS staining showed typical signs of injury, including intensified renal tubule luminal dilation, brush edge fractures, and tubule accumulation in CLP-treated WT mice, which were substantially more pronounced in the Sirt5CKO + CLP group (Figure 6D–E). Following CLP, protein levels of the kidney injury markers NGAL and KIM-1 were significantly more elevated in Sirt5CKO mice than in WT mice (Figure 6F, I–J). Meanwhile, compared to WT + CLP mice, CLP induced a significant increase in the content of BUN and Scr (Figure 6G–H) in Sirt5CKO mice, indicating the development of AKI. CLP-induced GSH depletion and MDA accumulation were significantly aggravated in the kidneys of Sirt5CKO mice compared with WT mice (Figure 6K and L). Additionally, Sirt5CKO mice experienced higher expression of HMOX-1 and NQO-1 proteins compared to WT mice after 24 hours of CLP (Figure 6M–O), and the Sirt5CKO + CLP group had increased content of Fe^2+^in kidneys (Figure 6P). The Sirt5CKO + CLP group showed more profound suppression of GPX4 protein and a more dramatic upregulation of ACSL4 expression, compared to the WT + CLP group (Figure 6Q–S).

### SIRT5 activation by MC3138 alleviated acute kidney injury *in vivo* model of SA-AKI

**Figure 7.**
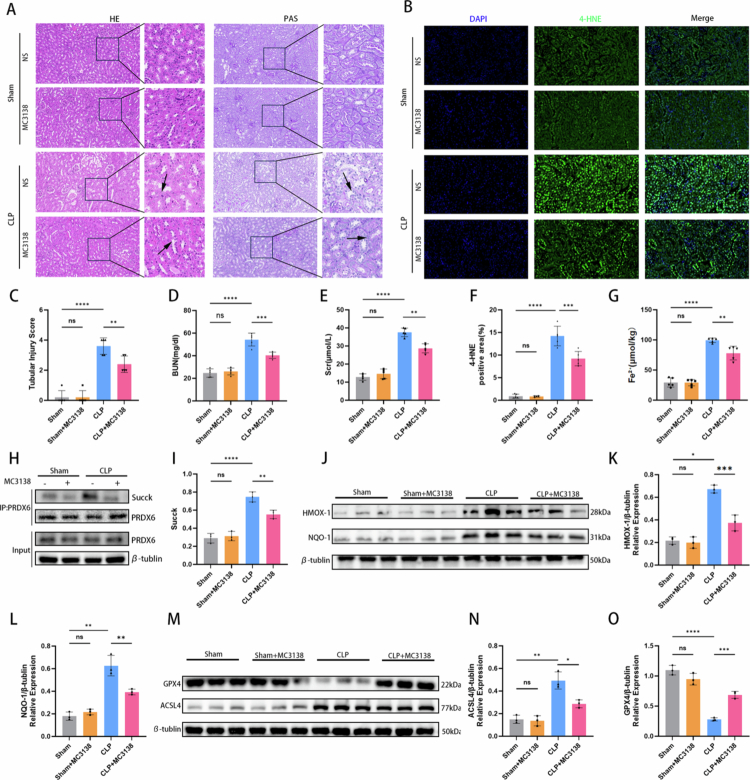
SIRT5 activation by MC3138 alleviated acute kidney injury in an *in vivo* model of SA-AKI. (A) Representative images of H&E and PAS staining of the kidney cortex in the Sham, Sham + MC3138, CLP, and CLP + MC3138 groups. (B)Immunofluorescence staining of 4-HNE (green) in the kidney tissues of the four groups; DAPI (blue) was used for nuclear staining. (C) Tubular injury scores based on H&E and PAS staining in four groups (*n* = 5). (D–E) Serum levels of BUN and Scr were measured at 24 hours after CLP surgery (*n* = 5 mice per group). (F) Quantitative analysis of 4-HNE positive area fraction (*n* = 5). (G) Bar graph shows the Fe^2+^ content of kidney homogenates in four groups (*n* = 5). (H–I) Immunoprecipitation (IP) and western blot analysis of PRDX6 succinylation levels in the kidneys (*n* = 3). (J–L) Western blotting results showing protein expression levels of HMOX1 and NQO-1 in kidney homogenates. Bar graphs show relative protein expression (*n* = 3). (M–O) Western blotting results showing protein expression levels of GPX4 and ACSL4 in kidney homogenates. Bar graphs show relative protein expression (*n* = 3). Data are presented as mean ± SD. Statistical significance is indicated as *P < 0.05, **P < 0.01, ***P < 0.001, ****P < 0.0001; ns, not significant. One-way ANOVA with Tukey test analysis and two-tailed Student's *t* test were used for statistical analysis.

To further evaluate the therapeutic potential of targeting SIRT5 in SA-AKI, the specific SIRT5 agonist MC3138 was administered to mice prior to CLP surgery. H&E and PAS staining revealed that CLP induction caused typical renal tubular injury, including luminal dilation and brush border disruption, whereas MC3138 treatment significantly ameliorated these histopathological changes (Figure 7A and C). Renal lipid peroxidation was assessed by detecting 4-hydroxynonenal (4-HNE) levels. Minimal 4-HNE-positive fluorescence was observed in the Sham and Sham + MC3138 groups, whereas the 4-HNE-positive area was significantly increased after CLP induction. Notably, MC3138 treatment markedly reduced 4-HNE accumulation in the renal tissues of CLP mice (Figure 7B and F), confirming that SIRT5 activation suppresses renal lipid peroxidation in SA-AKI. Consistently, serum levels of blood urea nitrogen (BUN) and creatinine (Scr) were markedly elevated in the CLP group compared with the Sham group, while MC3138 administration significantly reduced CLP-induced upregulation of BUN and Scr (Figure 7D–E), indicating that SIRT5 activation effectively improved renal function in SA-AKI mice. Additionally, MC3138 treatment reversed CLP-induced Fe^2+^ elevation in kidneys (Figure 7G). CLP induced a significant increase in PRDX6 succinylation without affecting total PRDX6 protein expression, and MC3138 treatment effectively reduced PRDX6 succinylation in SA-AKI mice (Figure 7H and I). Furthermore, CLP-induced upregulation of HMOX1, NQO-1, and ACSL4, as well as downregulation of GPX4, were all significantly reversed by MC3138 treatment (Figure 7J–O). Collectively, these results from the pharmacological intervention experiment further support SIRT5 as a therapeutic target for SA-AKI.

### The role of SIRT5-mediated protein desuccinylation in the development of SA-AKI

**Figure 8.**
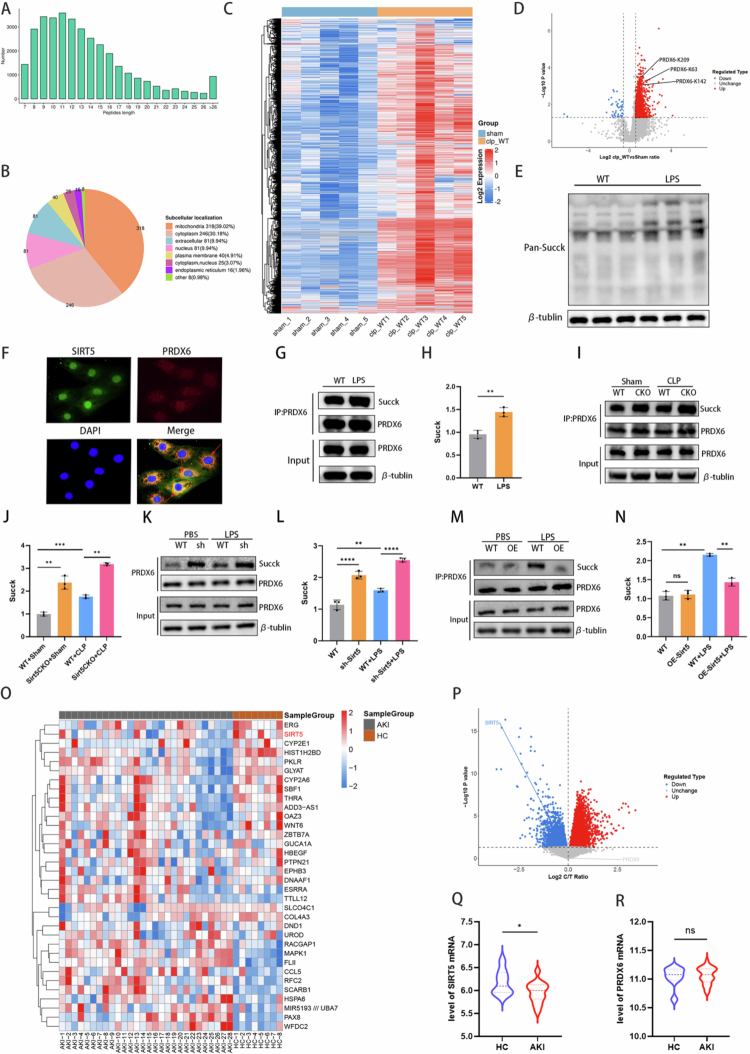
The role of SIRT5-mediated protein desuccinylation in the development of SA-AKI. (A) Distribution of tryptic peptide lengths. (B) Subcellular localization of identified succinylated proteins. (C) Heat map of differentially succinylated modification sites between the Sham and WT + CLP groups (*n* = 5 mice per group). (D) Volcano plot of differentially succinylated modification sites between the Sham and WT + CLP groups. (E) Total protein succinylation levels in HK-2 cells after 12 hours of LPS treatment in the WT and LPS groups. (F) Representative fluorescence images showing colocalization of SIRT5 (green) and PRDX6 (red) in HK-2 cells. Bright red arrows highlight specific sites of spatial colocalization. Colocalization was quantified by Pearson's correlation coefficient (PCC = 0.72, *n* = 5). (G–H) Expression and statistical analysis of succinylated PRDX6 protein in HK-2 cells after 12 hours of LPS treatment (*n* = 3). (I–J) Expression and statistical analysis of succinylated PRDX6 protein in kidney tissues of WT and Sirt5CKO mice at 24 hours after CLP (*n* = 3). (K–L) Expression and statistical analysis of succinylated PRDX6 protein in negative control (NC) and sh-Sirt5 HK-2 cells after 12 hours of LPS treatment (*n* = 3). (M–N) Expression and statistical analysis of succinylated PRDX6 protein in negative control (NC) and OE-Sirt5 HK-2 cells after 12 hours of LPS treatment (*n* = 3). (O) Unsupervised hierarchical clustering heatmap of differentially expressed genes (DEGs) between AKI patients and healthy controls (HC). (P) Volcano plot of DEGs between AKI patients and HC. (Q) Violin plot of SIRT5 mRNA expression in AKI and HC groups. (R) Violin plot of PRDX6 mRNA expression in AKI and HC groups. Data are presented as mean ± SD. Statistical significance is indicated as *P < 0.05, **P < 0.01, ***P < 0.001, ****P < 0.0001; ns, not significant. One-way ANOVA with Tukey test analysis and two-tailed Student's *t* test were used for statistical analysis.

To investigate SIRT5-mediated protein desuccinylation in SA-AKI, succinylation modification sites in kidney tissues of WT and WT + CLP mice were systematically analyzed by LC–MS/MS. Most peptide lengths were within the anticipated range of 8 to 20 amino acids, corresponding to tryptic peptides and confirming that sample preparation met proteomics analysis criteria (Figure 8A). Subcellular localization analysis revealed that numerous succinylated proteins were localized in the cytoplasm and mitochondria (Figure 8B). Quantitative data with a ratio > 1.5 or < 0.667 and a *P-*value < 0.05 were deemed to demonstrate differential succinylation for significantly differentially expressed proteins (DEPs). A heat map displayed the differentially succinylated modification sites between the WT and WT + CLP groups (Figure 8C), and a volcano plot showed these sites between the two groups (Figure 8D). Pan-succinylation analysis revealed that the total succinylation level of proteins in HK-2 cells was increased following LPS treatment (Figure 8E). Among the differentially succinylated modification sites, the succinylation level of PRDX6, a key enzyme involved in ferroptosis, was significantly upregulated. To explore the molecular mechanism by which SIRT5 regulates PRDX6 desuccinylation, the localization of SIRT5 and PRDX6 was examined. Immunofluorescence analysis showed that SIRT5 colocalized with PRDX6 (Figure 8F). Quantitative analysis revealed a high degree of colocalization between the two proteins, with a Pearson's correlation coefficient (PCC) of 0.72. Furthermore, LPS treatment induced a significant increase in PRDX6 succinylation without affecting total PRDX6 protein levels, paralleling the reduction in SIRT5 protein expression (Figure 8G and H). SIRT5 ablation markedly increased PRDX6 succinylation in both *in vivo* and *in vitro* SA-AKI models, with no effect on PRDX6 protein expression (Figure 8I–L). Conversely, PRDX6 succinylation was significantly reduced in the OE-Sirt5 + LPS group compared with the NC + LPS group (Figure 8M and N).

To validate the clinical relevance of the SIRT5-PRDX6 axis, a retrospective analysis of public transcriptomic datasets from the Gene Expression Omnibus (GEO) database was performed. Bioinformatic analysis revealed distinct gene expression profiles between AKI patients and healthy controls (HC). Unsupervised hierarchical clustering of differentially expressed genes (DEGs) clearly separated AKI samples from HC samples (Figure 8O). Consistently, volcano plot analysis identified widespread gene expression dysregulation in AKI, with SIRT5 being significantly downregulated while PRDX6 remained unchanged (Figure 8P). Violin plot analysis further demonstrated that SIRT5 mRNA expression was significantly decreased in AKI patients compared with HC (Figure 8Q), whereas PRDX6 mRNA expression showed no significant difference between the two groups (Figure 8R). These clinical dataset results are highly consistent with our *in vitro* and *in vivo* findings, confirming that SIRT5 downregulation is a key feature of AKI, while PRDX6 expression remains unchanged.

### SIRT5 desuccinylated PRDX6 at lysine 209 to weaken ferroptosis and alleviate kidney injury

**Figure 9.**
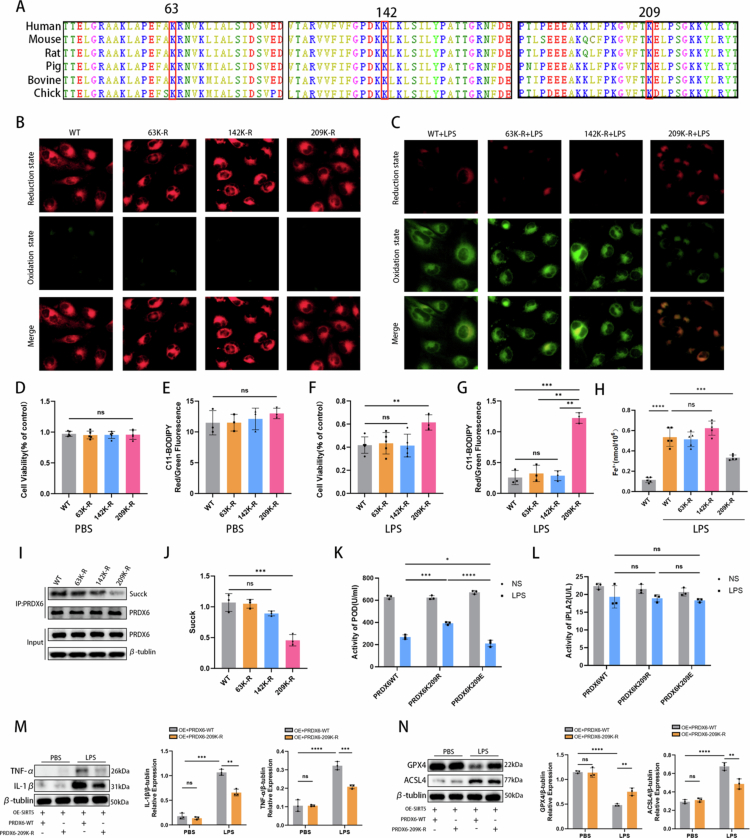
SIRT5 desuccinylated PRDX6 at lysine 209 to weaken ferroptosis and alleviate kidney injury. (A) The K63, K142, and K209 sites are highly conserved across different species, ranging from mice to humans. (B, E, C, G) Lipid ROS levels in cells transfected with different plasmids were visualized under an inverted fluorescence microscopy using the BODIPY 581/591 C11 probe. (D) Cell viability of HK-2 cells after transfection with different plasmids under PBS treatment (*n* = 5). (F) Cell viability of HK-2 cells after transfection with different plasmids under LPS treatment (*n* = 5). (H) Cellular Fe^2+^ content in five groups (*n* = 5). (I–J) Expression of and statistical analysis of succinylated PRDX6 protein in HK-2 cells after transfection with different plasmids. Bar graphs show relative protein expression (*n* = 3). (K) Peroxidase activity in three groups of HK-2 cells with or without LPS treatment (*n* = 3). (L) iPLA₂ activity in three groups of HK-2 cells with or without LPS treatment (*n* = 3). (M–N) Western blotting results showing the protein expression levels of IL-1β and TNF-*α* in four groups. Bar graphs show relative protein expression (*n* = 3). Data are presented as mean ± SD. Statistical significance is indicated as *P < 0.05, **P < 0.01, ***P < 0.001, ****P < 0.0001; ns, not significant. One-way ANOVA with Tukey's test analysis and two-tailed Student's *t* test were used for statistical analysis.

Succinylation proteomic analysis demonstrated that the Sham group and the WT + CLP group showed a significant difference in LysK63, LysK142, and LysK209 sites of PRDX6 (Figure 8D). These lysine residues are highly conserved across species from human to chick (Figure 9A). To investigate their functional roles, mutant plasmids (K63R, K142R, and K209R) were generated to mimic a desuccinylated state and transfected into HK-2 cells. Among these, K209 was identified as the primary succinylation site of PRDX6, as the K209R mutation resulted in a substantially reduced succinylation level (Figure 9I, J). Under basal conditions, no differences in cell viability or lipid peroxidation levels were observed among HK-2 cells transfected with PRDX6-WT, PRDX6-K63R, PRDX6-K142R, or PRDX6-K209R plasmids (Figure 9B, D, and E). Following LPS stimulation, cells transfected with PRDX6-K209R exhibited significantly higher cell viability and lower lipid peroxidation levels than the other three groups (Figure 9C, F, and G). Additionally, Fe²⁺ content was also reduced following the K209R mutation (Figure 9H). To clarify which enzyme activity of PRDX6 is affected by K209 modification, in vitro enzyme activity assays were performed. HK-2 cells were transfected with PRDX6-WT, PRDX6-K209R, or PRDX6-K209E (mimicking a permanently succinylated state) mutant plasmids. Under basal conditions without LPS stimulation, peroxidase activity was comparable among the three groups. Notably, LPS treatment significantly compromised peroxidase activity in PRDX6-WT-transfected cells. However, the K209R mutation markedly preserved this activity, whereas the K209E mutation led to a further decline in peroxidase function under LPS stress (Figure 9K). In contrast, iPLA₂ activity remained unchanged across all transfection groups, indicating that succinylation at K209 specifically modulates the peroxidase domain without affecting the phospholipase domain of PRDX6 (Figure 9L).

**Figure 10.**
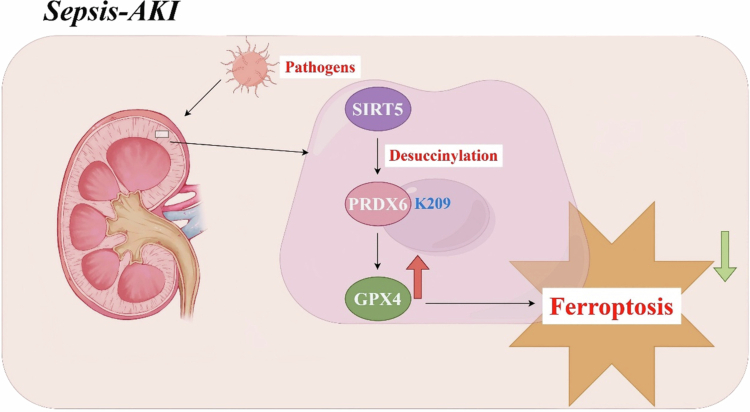
Schematic diagram illustrating the mechanism by which SIRT5-mediated desuccinylation of PRDX6 inhibits ferroptosis in the pathogenesis of SA-AKI.

Subsequently, PRDX6-WT and PRDX6-K209R plasmids were transfected into SIRT5-overexpressing HK-2 cells. In OE-SIRT5 HK-2 cells, the PRDX6-K209R + LPS group showed significantly reduced TNF-*α* and IL-1β expression compared with the PRDX6-WT + LPS group (Figure 9M). In addition, GPX4 protein expression was significantly restored, and ACSL4 protein expression was partially downregulated following PRDX6-K209R transfection (Figure 9N). Collectively, these findings further indicate that lysine 209 of PRDX6 is the critical residue through which SIRT5-mediated desuccinylation exerts a protective effect in the pathogenesis of SA-AKI (Figure 10).

## Discussion

SA-AKI is characterized by high incidence and mortality, and effective therapeutic options remain limited [23,24]. Therefore, precise and effective therapeutic targets are urgently needed. The pathogenesis of SA-AKI involves a complex interplay of inflammatory cascades, immune dysregulation, mitochondrial dysfunction, and microcirculatory disturbances induced by microvascular abnormalities. SIRT5, a member of the sirtuin family, regulates multiple critical biological processes, including oxidative stress, cell death, and mitochondrial function, and has been implicated in various diseases such as cancer, inflammation, and metabolic syndromes [25,26]. Notably, SIRT5 has been reported to be involved in sepsis-induced lung injury and diquat-induced renal tubular cell damage [16,27]; however, its specific role and underlying mechanisms in SA-AKI remain largely unexplored. In this study, we demonstrated that SIRT5 expression was significantly decreased in both cellular and animal models of sepsis. Loss of SIRT5 exacerbated ferroptosis in renal tubular epithelial cells, thereby promoting SA-AKI progression. Mechanistically, we further revealed that SIRT5 inhibited succinylation of PRDX6 at lysine 209. PRDX6 is a key enzyme responsible for detoxifying lipid peroxides, and this desuccinylation enhanced its enzymatic activity, ultimately suppressing ferroptosis. Collectively, this study identifies the SIRT5-PRDX6 axis as a critical regulator in SA-AKI and provides a potential therapeutic target for sepsis-induced renal injury.

Lysine succinylation is a newly identified post-translational modification that functions as a regulatory mechanism modulating mitochondrial metabolism and certain extramitochondrial biological processes [28]. It plays a critical role in diverse metabolic pathways, including fatty acid metabolism, the tricarboxylic acid (TCA) cycle, and amino acid degradation [29]. Among the known desuccinylases, SIRT5 is the most extensively studied and exerts pivotal effects on innate immune and inflammatory responses by modulating the succinylation levels of mitochondrial proteins [30]. Zhang et al. reported that in a sepsis model, downregulation of SIRT5 in macrophages elevates succinylation of TANK-binding kinase 1 (TBK1), which subsequently impairs its interaction with interferon regulatory factor 3 (IRF3), leading to reduced expression of proinflammatory cytokines and chemokines and thereby attenuating inflammation [31]. Moreover, SIRT5 has been reported to play a protective role in sepsis-induced lung injury. In an LPS-induced lung epithelial injury model, SIRT5 promoted desuccinylation of Homeobox A5, which subsequently activated transcription of ferroptosis suppressor protein 1 (FSP1), alleviating LPS-induced ferroptosis and epithelial cell injury [27]. In the present study, we observed a significant increase in global protein succinylation levels in HK-2 cells following LPS stimulation, accompanied by a marked decrease in SIRT5 expression. Furthermore, in mice with renal tubular epithelial cell-specific knockout of SIRT5, protein succinylation levels in the kidney were significantly elevated after CLP compared with wild-type controls, along with more severe tubular epithelial injury. These findings suggest that protein succinylation plays a critical role in the pathogenesis of SA-AKI. Targeting protein succinylation or modulating SIRT5 may therefore represent a promising therapeutic strategy to mitigate sepsis-induced kidney injury.

Infection and inflammation profoundly disrupt the antioxidant defense system, leading to iron-dependent lipid peroxide accumulation and glutathione depletion, which ultimately trigger ferroptosis and exacerbate pathological damage [32]. The kidney is considered one of the organs most sensitive to ferroptosis, as renal tubular epithelial cells are persistently exposed to a high-oxygen environment and are therefore more susceptible to oxidative stress [33]. Consequently, ferroptosis plays a critical role in septic injury to renal tubular epithelial cells. In the present study, high-throughput proteomic analysis revealed significant enrichment of differentially expressed proteins in the ferroptosis signaling pathway. Notably, the expression levels of ACSL4 and HMOX1, two key ferroptosis-promoting proteins, were markedly upregulated following CLP intervention. Furthermore, treatment with the ferroptosis inhibitor Fer-1 in an LPS-induced in vitro model of septic renal tubular epithelial injury significantly reduced the levels of the pro-inflammatory cytokines IL-6 and TNF-*α* compared with the model group, while also substantially improving cell viability. These findings underscore the pathogenic role of ferroptosis in sepsis-associated renal injury and suggest that targeting ferroptotic pathways may represent a potential therapeutic strategy.

PRDX6, a lipid hydroperoxide-detoxifying enzyme, is abundantly expressed in organs such as the lungs, brain, kidneys, and testes. It stands out among the peroxiredoxin family due to its unique combination of enzymatic activities [34,35]. PRDX6 possesses three distinctenzymatic functions: calcium-independent phospholipase A2 (iPLA2), lysophosphatidylcholine acyltransferase (LPCAT), and glutathione peroxidase (GPX)-like activity. These activities contribute to the repair of oxidized membrane phospholipids, making PRDX6 a critical negative regulator of ferroptosis [36]. Unlike GPX4, PRDX6 is not a member of the selenoprotein family; its peroxidase activity depends primarily on glutathione S-transferase. Despite this difference, PRDX6 and GPX4 exhibit a functional correlation. PRDX6 can bind to seleno-diglutathione, facilitating GPX4's utilization of selenium and enhancing their cooperative role in repairing oxidized phospholipids [37]. Emerging evidence has revealed that PRDX6 regulates ferroptosis and is involved in sepsis-induced multi-organ injury. Liang et al. reported that PRDX6 overexpression significantly inhibited LPS-induced ferroptosis in alveolar macrophages and attenuated lung injury in a CLP-induced sepsis-associated acute lung injury (SA-ALI) mouse model. Mechanistically, PRDX6 upregulates SLC7A11 expression by inhibiting p53, thereby enhancing cellular antioxidant capacity, mitigating lipid peroxidation and ferroptosis, and ultimately reducing inflammation and pulmonary damage [38]. In the present study, we identified an interaction between PRDX6 and SIRT5. Genetic depletion of SIRT5 increased succinylation of PRDX6, thereby impairing its enzymatic activity, whereas SIRT5 overexpression reversed this effect. Further LC–MS/MS analysis revealed a significant increase in succinylation at three lysine residues of PRDX6 (K63, K142, and K209) in mice with conditional knockout of SIRT5 in renal tubular epithelial cells. Mutagenesis analysis of these lysine residues demonstrated that K209 is the critical site for SIRT5-mediated desuccinylation. Importantly, the K209R mutant of PRDX6 significantly alleviated LPS-induced ferroptosis in renal tubular epithelial cells and mitigated tubular epithelial injury during sepsis. These findings highlight the pivotal role of SIRT5-regulated PRDX6 desuccinylation in modulating ferroptosis and renal protection under septic conditions.

This study has several limitations. First, the lack of direct human renal tissue samples from SA-AKI patients represents a major limitation. Although we observed downregulation of SIRT5 and its regulatory role in PRDX6 succinylation and ferroptosis in animal models and cell lines, the clinical translation of these findings cannot be fully confirmed without validation in human renal specimens. Second, the LPS-induced cell model and CLP-induced mouse model used to mimic SA-AKI may not fully recapitulate the complex pathophysiological environment of clinical sepsis. Moreover, *in vitro* experiments were performed using PRDX6 mutant plasmids, but adeno-associated virus-mediated intervention was not conducted in mice for *in vivo* verification. Future studies could establish PRDX6 K209R or K209E gene knockout mice to clarify the physiological function of this site *in vivo*. Third, this study focused on the K209 site of PRDX6; whether other SIRT5 substrates are involved in the regulation of ferroptosis in SA-AKI remains to be elucidated. Studies have shown that curcumin attenuates osteoarthritis progression by enhancing SIRT5-mediated desuccinylation of ACSL4, thereby inhibiting chondrocyte ferroptosis [39]. Another study demonstrated that quercetin-4′-O-*β*-D-glucoside alleviates diabetic nephropathy by upregulating SIRT5 expression, which promotes desuccinylation of TFR1 at lysine 626, reducing its protein stability and inhibiting ferroptosis [40]. Therefore, it is reasonable to speculate that SIRT5 may target other ferroptosis-associated proteins to exert synergistic protective effects in SA-AKI.

Despite these limitations, our data provide clear *in vitro* and *in vivo* evidence that SIRT5-mediated PRDX6 desuccinylation protects against septic renal tubular injury by inhibiting ferroptosis. Future studies using human clinical samples and more comprehensive succinylome analysis will help to further validate the clinical relevance of this mechanism and translate it into potential therapeutic strategies for SA-AKI.

Additionally, the molecular mechanism underlying SIRT5 downregulation in SA-AKI remains unresolved. Our preliminary qRT‒PCR data showed a significant reduction in SIRT5 mRNA levels in SA-AKI mouse renal tissues, suggesting that transcriptional repression may be a major contributor to its decreased expression. This may be driven by the binding of pro-inflammatory transcription factors (e.g. NF-κB, AP-1) to the SIRT5 promoter region under septic inflammatory stress. Further exploration of the transcription factors regulating SIRT5 expression will be the focus of our subsequent research. Alternatively, SIRT5 protein stability may be impaired in SA-AKI via ubiquitin-proteasome or autophagic degradation pathways. This open question provides a critical direction for future research to fully elucidate the SIRT5-mediated anti-ferroptosis axis in SA-AKI, which will help identify more upstream targets for therapeutic intervention and enrich our understanding of the epigenetic regulation of SA-AKI.

In summary, this study demonstrates that SIRT5 acts as a critical mediator in the progression of SA-AKI through the regulation of ferroptosis and oxidative stress. The protective role of SIRT5 in SA-AKI depends on its ability to enhance the peroxidase activity of PRDX6 by modulating its succinylation level, which restores the lipid peroxidation imbalance induced by sepsis in renal tubular epithelial cells and thereby alleviates cellular injury.

## Supplementary Material

Author Checklist.pdf Author Checklist.pdf

S1.docx S1.docx

## Data Availability

The data that support the findings of this study are available from the corresponding author upon reasonable request.
