# Supplementary material for: Sirtuin 5‐mediated desuccinylation of PRDX6 inhibits ferroptosis and alleviates sepsis-associated acute kidney injury
Source: Redox Rep. 2026 Apr 10;31(1):2657075. doi: 10.1080/13510002.2026.2657075 (PMC13072693; doi:10.1080/13510002.2026.2657075)
Supplement: S1.docx [file YRER_A_2657075_SM2593.docx]

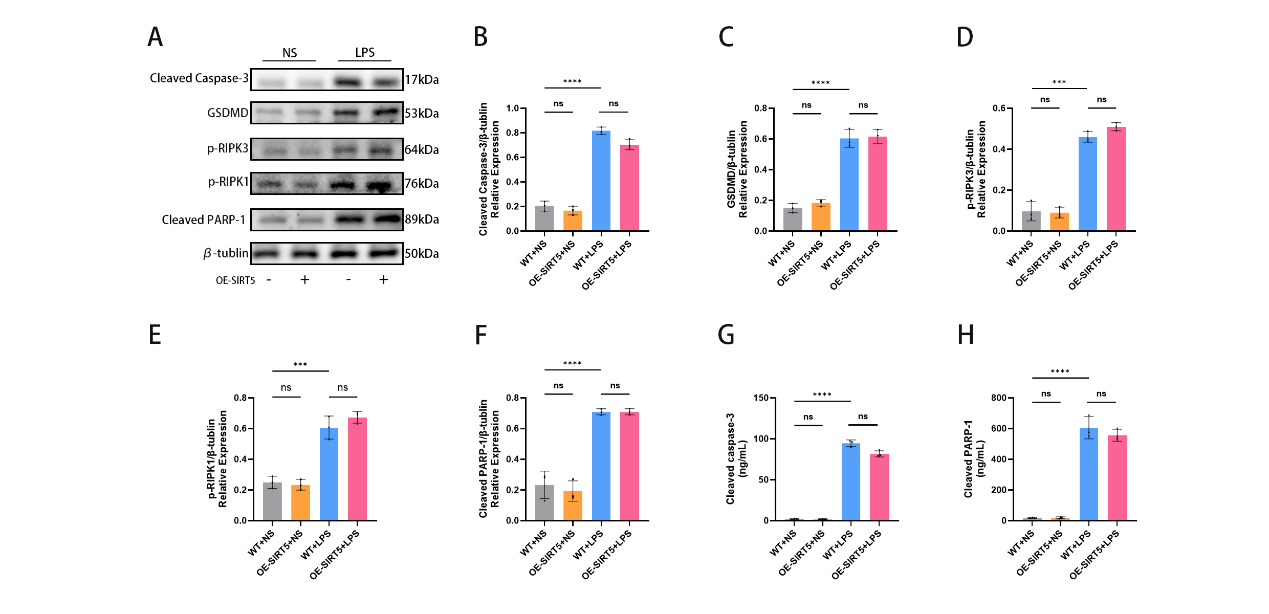


Fig. S1 (A) Western blotting results show the protein expression levels of Cleaved Caspase-3, GSDMD, p-RIPK3, p-RIPK1, and Cleaved PARP-1 in HK-2 cells with or without SIRT5 overexpression (OE-SIRT5) under control (NS) or LPS-stimulated conditions(n = 3). (B–F) Quantitative analysis of relative protein expression levels of Cleaved Caspase-3 (B), GSDMD (C), p-RIPK3 (D), p-RIPK1 (E), and Cleaved PARP-1. Bar graphs show the protein relative expression(n = 3). (G–H) ELISA quantification of Cleaved Caspase-3 and Cleaved PARP-1 levels in cell lysates(n = 3). Data are presented as mean ± SD. Statistical significance is indicated as **P* < 0.05, ***P* < 0.01,*** *P* <0.001, **** *P* <0.0001; ns, not significant. One-way ANOVA with Tukey test analysis and two-tailed Student’s *t* test were used for statistical analysis.
